# Contraceptive implant migration to the ulnar nerve: A case report with literature review

**DOI:** 10.1002/ccr3.9420

**Published:** 2024-09-03

**Authors:** Saywan K. Asaad, Nigar M. Salih, Marwan N. Hassan, Mohammed S. Abid, Hawbash F. Hamid, Nahidah H. Ameen Ahmed, Huda M. Muhammad, Abdullah K. Ghafoor, Snur Othman, Fahmi H. Kakamad

**Affiliations:** ^1^ College of Medicine University of Sulaimani Sulaimani Kurdistan Iraq; ^2^ Smart Health Tower Sulaimani Kurdistan Iraq; ^3^ Sulaimani maternity teaching hospital Sulaimani Kurdistan Iraq; ^4^ Kscien Organization Sulaimani Kurdistan Iraq

**Keywords:** Implanon, neuropathy, Nexplanon, ulnar nerve

## Abstract

**Key Clinical Message:**

Contraceptive implant migration is a rare complication associated with contraceptive implants: migration to the ulnar nerve, emphasizing the importance of accurate diagnosis, imaging, and a multidisciplinary approach to mitigate neurovascular risks during insertion and removal procedures. The case report demonstrates the necessity for careful removal techniques and thorough patient follow‐up to ensure positive outcomes and prevent long‐term nerve damage.

There are some potential risks and complications associated with contraceptive implants, including neurovascular injury. The aim of this case report is to report a rare complication associated with contraceptive implants. A 32‐year‐old female, right‐hand dominant, presented to the orthopedic clinic for the extraction of a contraceptive implant (Implanon) from her left arm. She reported intermittent numbness in the ring and little fingers. Upon examination, the Implanon was not palpable. Both Phalen's test and Tinel signs were negative. An x‐ray of the arm revealed the implant's position. Under local anesthesia through a longitudinal incision, the Implanon was found within the perineurium of the ulnar nerve. Two weeks after the operation, the patient returned to the clinic. Upon examination, there were no indications of ulnar nerve neuropathy. If a patient undergoes subdermal implant‐associated pain or is at risk of neurovascular damage during removal, it is advisable to refer the patient to a family planning specialist experienced in handling challenging implant removals, and subsequently to a peripheral nerve surgeon, to optimize outcomes. The migration of a contraceptive implant to the ulnar nerve is an exceedingly rare but possible complication.

## 1 INTRODUCTION

Contraceptive implants are a safe and effective form of long‐acting reversible contraception (LARC) that provides up to 3–5 years of protection against pregnancy.^1^, ^2^ The insertion and removal processes are tailored for safe execution in outpatient setting using local anesthesia. They are implanted just beneath the skin of the upper arm and dispense a little dose of the progestin hormone to inhibit ovulation.^3^ It has a failure rate of <1% per year.^4^ While contraceptive implants are generally very safe, there are some potential risks and complications associated with their use, including neurovascular injury.^5^ This complication was frequently observed in earlier devices, particularly when inserted deeply or experienced proximal migration. Several case studies have documented injuries related to the implant, affecting the median, ulnar, and medial antebrachial cutaneous nerves.^1^, ^2^, ^6^, ^7^ These findings led to technique‐technique device design modifications and implantation technique adjustments.^5^ One rare but serious complication is the migration of the implant to the ulnar nerve.^1^, ^4^ The ulnar nerve is a major nerve in the arm that provides sensation and motor function to the hand and forearm.^2^, ^4^ In severe cases, ulnar nerve compression can damage permanent nerve.^4^

The aim of this case report is to document and discuss a rare and unique complication associated with contraceptive implants—which is the migration of the implant to the perineurium of the ulnar nerve.

### 1.1 Case history/examination

A 32‐year‐old housewife, female, right‐hand dominant, referred by a gynecologist to the orthopedic clinic for the removal of a contraceptive implant from her arm. The implant had been inserted into her left arm 8 months earlier by a senior gynecologist. Initially, the patient could feel the implant for 3 months. She experienced an undesirable menstrual bleeding pattern and reported intermittent numbness in the ring and little fingers, and she had a negative medical history, with three previous cesarean sections. Upon examination, the Implanon was not palpable. Both Phalen's test and Tinel's signs yielded negative results. Her body mass index was 24.3 kg/m^2^.

### 1.2 Methods (differential diagnosis, investigations and treatment)

**FIGURE 1:**
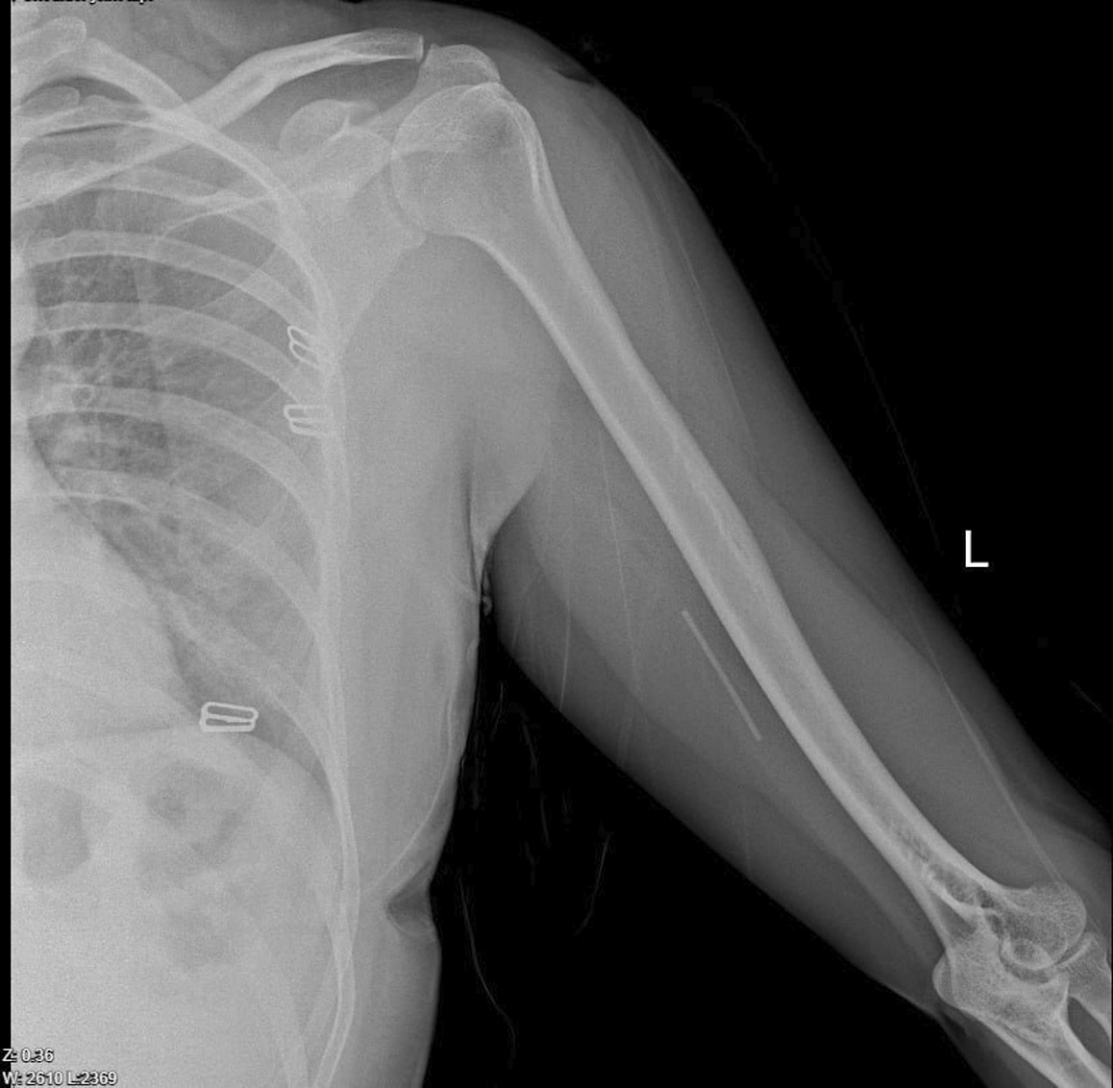
Pre‐operative x‐ray: Evidence of implant device deeply migrated.

**FIGURE 2:**
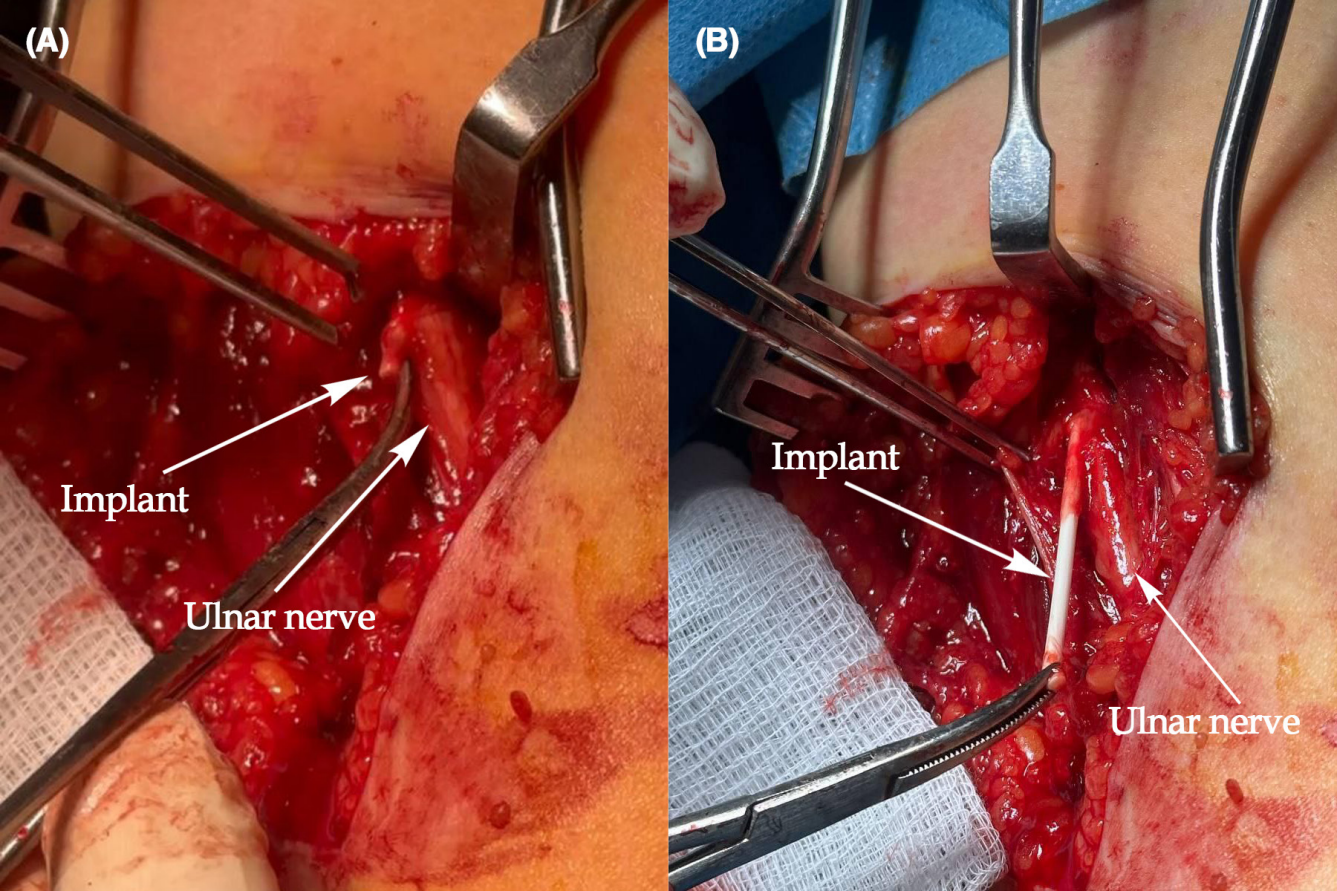
Contraceptive implant situated in the perineurium of the ulnar nerve.

An x‐ray of the arm revealed the implant's position, as depicted in Figure 1. Ultrasound examination did not identify any foreign bodies in her left arm. In the operating room, under local anesthesia and in a supine position, a 5 cm longitudinal incision was made in the medial aspect of the distal left arm. The Implanon was found within the perineurium of the ulnar nerve (Figure 2). Careful removal of the Implanon was performed without damaging the ulnar nerve. Hemostasis was secured, and the incision was closed in layers.

### 1.3 Conclusion and results (outcome and follow‐up)

The patient was discharged home 2 h postoperatively. Two weeks after the operation, the patient returned to the clinic for suture removal. She was in good condition, no signs of infection, and the stitches were removed. Upon examination, there were no signs of ulnar nerve neuropathy.

## 2 DISCUSSION

The use of contraceptive implants comes with its set of challenges and potential complications.^3^ The insertion and removal of subdermal contraceptive implants are typically quick office procedures conducted under local anesthesia.^6^ The implant removal occurs after 3 years or earlier if requested by the patient. Patients may opt for removal due to diverse reasons such as weight gain, a desire to become pregnant, or encountering an undesired pattern menstrual of bleeding.^8^ Complications during insertion are infrequent but can include infection, migration, expulsion, hematoma, and allergic reactions.^1^ While earlier studies indicated a migration rate as high as 39% among patients, a recently conducted study involving 4294 practitioners revealed a migration incidence of merely 0.26%.^9^, ^10^ Typically, migration does not exceed 2 cm from the initial insertion site.^9^, ^10^

Inserting or removing a contraceptive implant into the arm poses a potential risk to peripheral nerves. Ulnar neuropathy is an exceedingly rare consequence in the genuine literature.^1^, ^11^ The ulnar nerve arises from the brachial plexus, encompassing fibers from the C8‐T1 nerve roots. Its course extends along the medial aspect of the upper extremity. Around the midpoint of the arm, it penetrates the fascia, following the path along the medial head of the triceps muscle. Progressing distally along the posterior facet of the medial epicondyle, it enters the forearm between the heads of the flexor carpi ulnaris. Subsequently, it traverses deep along the ulna, where it bifurcates into its muscular, palmar cutaneous, and dorsal cutaneous branches.^12^

Two types of peripheral neurological complications have been documented: those linked to compressive neuropathy due to device pressure during insertion and complications from improper device removal. Acute peripheral neuropathy related to contraceptive insertion is an even rarer complication associated with an excessive injection angle.^2^ Due to safety concerns, healthcare professionals must undergo training and certification mandated by the Food and Drug Administration before integrating contraceptive implants into their clinical practice.^5^, ^7^ Before 2020, practitioners were advised to insert the implant beneath the dermis within the sulcus between the biceps and triceps muscles. Due to challenges in removal and the potential for migration, the insertion site was modified in 2020 to be situated over the triceps muscle, approximately 8–10 cm above the medial epicondyle and 3–5 cm posterior to the sulcus between the biceps and triceps muscles.^8^ The latest guidelines from the manufacturer advise against inserting the implant in the sulcus between the biceps and triceps, where the median nerve, ulnar nerve, brachial artery, and vein are situated.^8^ While the focus is typically on preventing excessively deep placement, shallow implantation can also lead to issues.^13^ Placing the implant between the epidermis and dermis poses the risk of exposure and persistent painful stimulation to sensory receptors and nerves in this layer.^13^

If a patient undergoes subdermal implant‐associated pain or is at risk of neurovascular damage during removal (indicated by the implant's deep location to muscle fascia or proximity to a neurovascular structure as observed during a physical exam or imaging), it is advisable to refer the patient to a family planning specialist experienced in handling challenging implant removals, and subsequently to a peripheral nerve surgeon, to optimize outcomes.^13^ Indicators of an iatrogenic nerve injury during placement or removal include electric or shock‐like pain, numbness, or weakness within the distribution of a peripheral nerve. Physical examination may reveal reduced sensitivity to touch and weakness in the hand or forearm. Left untreated, late signs of nerve injury may manifest as evident muscle wasting or abnormal posturing, such as the ulnar claw hand.^1^, ^5^ In this consulted‐consulted case; the patient is sensing numbness in the ring and little fingers without consulting a doctor to identify the cause. She intended to have the implant removed because of experiencing an undesirable bleeding pattern.

Given the potential for deep implantation and migration, imaging studies are recommended to accurately pinpoint migrated or non‐palpable devices before removal. In a study detailing the operative removal of 28 contraceptive devices, it was found that 30% of implants had migrated from the insertion site, with 37% located intramuscularly and 11% within the neurovascular sheath.^14^ Additionally, a study strongly aligns with the manufacturer's suggestion that attempting extraction without precise knowledge of the device's location should be avoided.^5^ For any patient displaying neurological symptoms, it is advisable to conduct imaging studies. X‐ray, CT, MRI, and ultrasound are all viable options for accurately determining the location of an implant. X‐ray and ultrasound are cost‐effective, accessible, and non‐invasive imaging modalities. Ultrasound is a non‐invasive imaging technique that utilizes high‐frequency sound waves to create real‐time images of internal structures within the body.^2^, ^5^, ^15^ Prompt and accurate diagnosis and treatment are imperative to safeguard treatment choices and optimize patient outcomes. It's essential to note that delayed or incomplete management of nerve injuries, especially those induced iatrogenically, can lead to significant legal liabilities.^5^

This study underscores the importance of employing a multidisciplinary approach when addressing complications linked to contraceptive implants, especially in cases involving subdermal implant migration, associated pain, or the potential for neurovascular damage during extraction. In such scenarios, it is recommended to direct patients to an orthopedic surgeon for optimal outcomes. This approach aims to minimize complications and ensure the protection of the patient's neurological health. Timely and accurate interventions, informed by thorough clinical examination and imaging studies, are crucial for the successful removal of contraceptive implants. This collaborative approach elevates the overall standard of care for individuals grappling with uncommon complications such as migration to the ulnar nerve.

Osman et al. reported a young woman experiencing ulnar nerve paraesthesia after insertion, which spontaneously resolved.^16^ Saeed et al. documented a case where a woman presented with paraesthesia along the ulnar distribution of her hand and forearm 1 day after contraceptive implant insertion, along with shooting pain on palpating the ulnar nerve course. Ultrasonography revealed the implant positioned in the subfascial plane of the inner arm. Surgical removal later identified the implant within the perineurium of the ulnar nerve, resulting in ulnar nerve neuropathy.^4^ A recent comprehensive literature review found 63 papers describing implant migrations. This review specifically examined 12 patients with a total of 14 nerve injuries. Two injuries occurred during or before device insertion, while 12 happened during removal. The nerves predominantly affected were the medial antebrachial cutaneous and median nerves. The primary causes of nerve injury were inadvertent pulling or grasping of the nerve, mistaking it for the implant.^17^ In this case, the implant was meticulously identified and removed cautiously, ensuring no nerve injury occurred.

In conclusion, the migration of a contraceptive implant to the ulnar nerve is an exceedingly rare yet possible complication. The present study suggests documenting the exact location of the implant through meticulous physical examination and imaging studies prior to extraction.

## AUTHOR CONTRIBUTIONS

**Saywan K. Asaad:** Conceptualization; formal analysis; resources; validation; visualization; writing – original draft; writing – review and editing. **Nigar M. Salih:** Conceptualization; resources; validation; visualization; writing – review and editing. **Marwan N. Hassan:** Methodology; resources; validation; visualization; writing – review and editing. **Mohammed S. Abid:** Conceptualization; formal analysis; validation; writing – review and editing. **Hawbash F. Hamid:** Data curation; resources; validation; visualization; writing – review and editing. **Nahidah H. Ameen Ahmed:** Conceptualization; investigation; validation; visualization; writing – review and editing. **Huda M. Muhammad:** Conceptualization; data curation; investigation; validation; writing – review and editing. **Abdullah K. Ghafoor:** Data curation; formal analysis; investigation; methodology; writing – review and editing. **Snur Othman:** Conceptualization; investigation; resources; validation; visualization; writing – review and editing. **Fahmi H. Kakamad:** Conceptualization; validation; visualization; writing – review and editing.

## FUNDING INFORMATION

The authors have no funding to report.

## CONFLICT OF INTEREST STATEMENT

The authors declare no conflict of interest.

## ETHICS STATEMENT

Not applicable.

## CONSENT

Written informed consent was obtained from the patient to publish this report in accordance with the journal's patient consent policy.

## Data Availability

The data that support the findings of this study are available from the corresponding author upon reasonable request.
